# Chemokines as Prognostic Factor in Colorectal Cancer Patients: A Systematic Review and Meta-Analysis

**DOI:** 10.3390/ijms25105374

**Published:** 2024-05-15

**Authors:** Johanna Fellhofer-Hofer, Clemens Franz, Johannes A. Vey, Christoph Kahlert, Eva Kalkum, Arianeb Mehrabi, Niels Halama, Pascal Probst, Fee Klupp

**Affiliations:** 1 Department of General, Visceral and Transplantation Surgery, University of Heidelberg, Im Neuenheimer Feld 420, 69120 Heidelberg, Germany; johanna.fellhofer-hofer@med.uni-heidelberg.de (J.F.-H.); clemens.franz@med.uni-heidelberg.de (C.F.); christoph.kahlert@med.uni-heidelberg.de (C.K.); arianeb.mehrabi@med.uni-heidelberg.de (A.M.); pascal.probst@stgag.ch (P.P.); 2 Institute of Medical Biometry (IMBI), University Hospital Heidelberg, Im Neuenheimer Feld 130/3, 69120 Heidelberg, Germany; vey@imbi.uni-heidelberg.de; 3 Study Center of the German Society of Surgery (SDGC), University of Heidelberg, Im Neuenheimer Feld 130/3, 69120 Heidelberg, Germany; eva.kalkum@med.uni-heidelberg.de; 4 National Center for Tumor Diseases, Medical Oncology and Internal Medicine VI, Tissue Imaging and Analysis Center, Bioquant, University of Heidelberg, Im Neuenheimer Feld 267, 69120 Heidelberg, Germany; niels.halama@med.uni-heidelberg.de; 5 Helmholtz Institute for Translational Oncology (HI-TRON), Department of Cancer Immunology & Cancer Immunotherapy, German Cancer Research Center (DKFZ), 55131 Mainz, Germany; 6 Department of Surgery, Cantonal Hospital Thurgau, Pfaffenholzstrasse 4, 8501 Frauenfeld, Switzerland

**Keywords:** colorectal cancer, chemokine, CXCR4 expression, outcome, survival

## Abstract

Chemokines orchestrate many aspects of tumorigenic processes such as angiogenesis, apoptosis and metastatic spread, and related receptors are expressed on tumor cells as well as on inflammatory cells (e.g., tumor-infiltrating T cells, TILs) in the tumor microenvironment. Expressional changes of chemokines and their receptors in solid cancers are common and well known, especially in affecting colorectal cancer patient outcomes. Therefore, the aim of this current systematic review and meta-analysis was to classify chemokines as a prognostic biomarker in colorectal cancer patients. A systematic literature search was conducted in PubMed, CENTRAL and Web of Science. Information on the chemokine expression of 25 chemokines in colorectal cancer tissue and survival data of the patients were investigated. The hazard ratio of overall survival and disease-free survival with chemokine expression was examined. The risk of bias was analyzed using Quality in Prognosis Studies. Random effects meta-analysis was performed to determine the impact on overall respectively disease survival. For this purpose, the pooled hazard ratios (HR) and their 95% confidence intervals (CI) were used for calculation. Twenty-five chemokines were included, and the search revealed 5556 publications. A total of thirty-one publications were included in this systematic review and meta-analysis. Overexpression of chemokine receptor CXCR4 was associated with both a significantly reduced overall survival (HR = 2.70, 95%-CI: 1.57 to 4.66, *p* = 0.0003) as well as disease-free survival (HR = 2.68, 95%-CI: 1.41 to 5.08, *p* = 0.0026). All other chemokines showed either heterogeneous results or few studies were available. The overall risk of bias for CXCR4 was rated low. At the current level of evidence, this study demonstrates that CXCR4 overexpression in patients with colorectal cancer is associated with a significantly diminished overall as well as disease-free survival. Summed up, this systematic review and meta-analysis reveals CXCR4 as a promising prognostic biomarker. Nevertheless, more evidence is needed to evaluate CXCR4 and its antagonists serving as new therapeutic targets.

## 1. Introduction

Colorectal cancer (CRC) is the third most common malignancy in the world and is one of the deadliest malignant tumors. Worldwide, 935,000 deaths were stated in 2020 [1]. Appallingly, by 2030, about 2.2 million new cases of CRC and 1.1 million deaths owing to CRC are predicted [2].

Expressional changes in chemokines and their receptors are known to provoke and modulate solid cancers like pancreatic cancer, breast cancer, melanoma as well as colorectal cancer [3,4,5,6,7]. Approximately 50 different chemokines are known, divided into four groups. The classification depends on the four conserved cysteine residues [8]. The primary amino acid sequence and the structural arrangement of the cysteine residues are defining for the chemokine subfamily [9]. At the N-terminus of the chemokine ligand, the conserved cysteine amino acid residues are connected to each other via disulfide bridges [9]. Balanced chemokine expression is responsible for proper immune cell function [10]. Chemokines and their receptors are expressed by stromal, immune, epithelial-endothelium and mesenchymal cells, leading to facilitated intercellular communication [11]. Table 1 is intended to provide an overview of the chemokine receptors with a focus on CXCR and corresponding ligands.

In tumor cells, an imbalance between pro- and anti-apoptotic proteins triggered by chemokines can occur, allowing tumor cell survival, tumor growth and inhibition of apoptosis [13,14]. Chemokines are integrated into several cellular interactions, such as angiogenesis and hematopoiesis and in the intrinsic communication of the human immune system [15,16,17]. Famous examples include CXCL9 and CXCL10 as central regulators of T cell densities and composition of T cells in the tumor microenvironment [18,19,20]. However, even these chemokines have come under scrutiny in other tumor diseases, with evidence also pointing to a possible detrimental role in tumor progression [18,19,20]. Another relevant example includes the chemokine receptor CXCR4, which is expressed in various cells, including endothelial and hematopoietic cells, as well as embryonic and adult stem cells [21]. CXCR4 is the predominant receptor for the chemokine ligand CXCL12; overexpression of CXCR4 and CXCL12 is found in several solid cancers, such as CRC, gastric cancer, pancreatic cancer and lung cancer [22,23,24,25,26]. Nevertheless, a differentiation between various tumor types on the basis of chemokine panel is not possible. However, chemokines represent tumorigenic markers whose dysregulation is known to influence survival [23,24,26]. Chemokine receptor CXCR4 is a seven-transmembrane G-Protein-couple receptor, which interacts with the chemokine ligand 12 (CXCL12). CXCL12 is also called SDF-1 (Chemokine stromal cell-derived factor-1) which is expressed on stromal cells, including endothelial cells and fibroblasts [8,27]. Research has shown that CXCL12 and CXCR4 impact angiogenesis and tumor growth, as well as invasion and metastasis [8,28]. Khare et al. reported in their study that both the CXCL12-CXCR4 axis and the CXCL12-CXCR7 axis play an important role in CRC metastasis [29]. In addition to tumor metastasis, CXCR4 is also involved in the modulation of carcinogenic stem cells [30]. Regulation of apoptosis also occurs via the CXCL12-CXCR4 axis with the participation of members of the Bcl-2 family [31]. The transmission of epithelial and mesenchymal colon cancer cells and their metastases are promoted by the interaction of CXCL12-CXCR4, leading to a direct impact on the Wnt-ß-catenin signaling pathway, which contributes to the formation and progression of colorectal cancer [9,32]. In general, colorectal cancers exhibit a variety of potential underlying mutations and immune markers, e.g., BRAF, KRAS, APC, PD1, and microsatellite stability (MSS), resulting in impaired survival [33,34,35]. Chemokine receptor type 7 (CXCR7) is expressed in many cancer cells and controls angiogenesis, cell growth and immunity [29]. In addition, CXCR7 has a stimulating effect on tumor development [36]. CXCR4 and CXCR7 can be expressed either individually or together; homo- or hetero-dimers can be formed when CXCR4 and CXCR7 are expressed simultaneously [29]. The presence of heterodimers has been demonstrated in about 65% of colorectal cancers [29]. Interactions occur through intracellular signaling effectors when CXCR4 and CXCR7 bind to the chemokine ligand CXCL12 [29,37]. Clinical trial efforts are underway, targeting either CXCL12 or the receptor CXCR4 in order to improve antitumoral activation of the immune system [20,38]. Due to a large number of different chemokines and chemokine receptors, which were addressed in a large number of diverse studies, an investigation of the reliability and validity of chemokines and chemokine receptors as potential prognostic factors in colorectal cancer patients is needed. Consequently, the aim of our systematic review and meta-analysis was to investigate the prognostic role of chemokine expression in the tumoral tissue of patients with CRC in order to explore possible modular trends within the broad heterogeneity of chemokines. Systematic review and meta-analysis were performed for colorectal cancer only, justified due to the huge number of studies regarding chemokine expression in various solid cancer types. A systematic review and meta-analysis should be performed for each cancer entity itself.

## 2. Results

### 2.1. Study Selection

**Figure 1:**
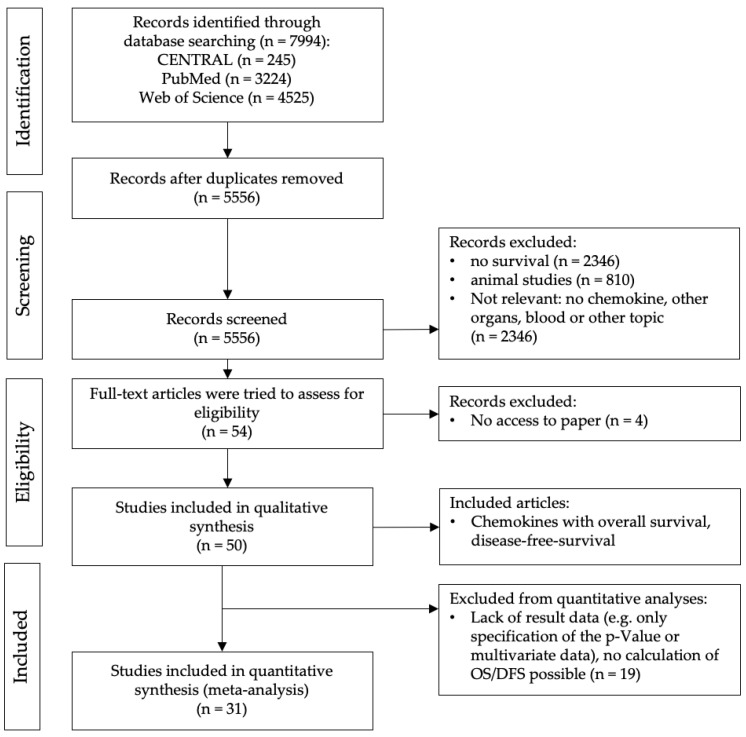
Prisma Flow Chart: showing the selection process.

Initial search revealed a total number of 7994 articles. Of these, 2438 duplicates were removed due to exclusion criteria. The titles and abstracts of 5556 articles were screened for inclusion. Records with no survival, animal studies or non-relevant were excluded. Fifty-four papers were actually included by screening the title and abstract. Four papers were excluded due to lack of access to these papers. A total of 50 articles were assessed for eligibility and met the inclusion criteria. Another 19 studies showed too little data on survival and were excluded too. Finally, the remaining 31 papers were included in the quantitative analysis, in which the survival of 6079 patients with chemokine expression in tumor tissue was analyzed. In Figure 1, a PRISMA flow chart is presented. All included and evaluated studies are shown in Table 2.

### 2.2. Qualitative Analysis

Study participation

A total of 3 out of 31 studies (9.7%) were at moderate risk, and the majority twenty-eight of thirty-one studies (90.3%) were at a low risk.

Study attrition

Eight out of thirty-one studies (25.8%) were at a moderate risk of bias, and the majority, 23 of the 31 studies (74.2%), were at a low risk of bias.

Prognostic factor measurement

All 31 studies (100%) received a low risk of bias score.

Outcome measurement

Three of thirty-one studies (9.7%) were at a moderate risk of bias. The majority, 28 of the 31 studies (90.3%), were at a low risk of bias.

Study confounding

Nine of the thirty-one studies (29%) were at a moderate risk of bias. A total of 22 of the 31 studies (71%) were at a low risk of bias.

Statistical analysis and reporting

A total of 8 of the 31 studies (25.8%) were at moderate risk of bias. The majority, 23 of the 31 studies (74.2%), were at a low risk of bias.

An overview of the risk of bias evaluated with the QUIPS tool is given in Table 2.

### 2.3. Study Characteristics

Additionally, detailed study information, such as the number of patients or AJCC as well as the year of publication, are presented in Table 3 and in Supplementary Tables S1A–D.

### 2.4. Quantitative Analysis of Chemokine Expression and Survival

A meta-analysis was only carried out if there were three or more studies available.

Quantitative analysis revealed that of the 25 assessed chemokines, only for chemokine CXCR4 significant results could be verified.

Four studies of the chemokine CXCL12 for overall survival and disease-free survival were included. One of the studies showed that increased expression led to better survival, contradicting the other three studies. Therefore, no significant result could be shown. Three studies were included on chemokine expression CXCL14 in relation to overall survival, which did not produce any significant results. Three studies on the chemokine CXCL1 were included; however, they were not pooled due to considerable heterogeneity, as recommended by the Cochrane Handbook. For the chemokine CXCL8, four studies on overall survival were included, which also showed broad heterogeneity. Chemokines for which three or more publications were included (CXCL1, CXCL8, CXCL12, CXCL14) data are shown in Supplementary Figure S1A–E.

For all other chemokines examined in this study, there were too few studies to obtain meaningful results.

**Figure 2:**
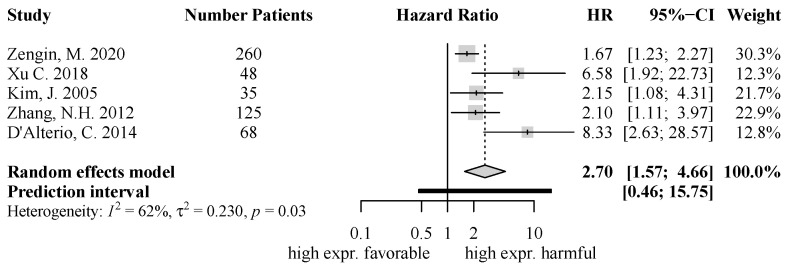
Forest Plot for CXCR4 for OS: HR = 2.70, 95% CI [1.57; 4.66], *p* = 0.0003; [22,36,46,48,56].

For CXCR4 systematic review and meta-analysis showed significant results for the chemokine with an influence on overall survival and disease-free survival. Five studies were included regarding CXCR4 expression and overall survival. All individual study results, as well as the pooled effect, showed that a tumoral overexpression of CXCR4 was associated with a significantly shortened overall survival (HR = 2.70, 95% CI [1.57; 4.66], *p* = 0.0003) (Figure 2). There was a moderate level of heterogeneity (I^2^ = 62%, τ^2^= 0.230).

**Figure 3:**
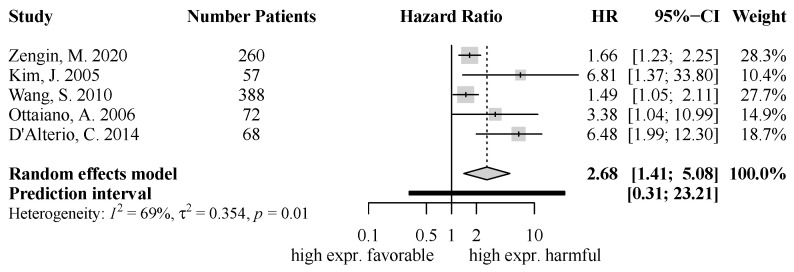
Forest Plot for CXCR4 for DFS: HR = 2.68, 95% CI [1.41; 5.08], *p* = 0.0026; [22,36,48,50,51].

Also, five studies were included regarding CXCR4 expression and disease-free survival. High CXCR4 expression in colorectal cancer specimens was accompanied by a significantly diminished disease-free survival (HR = 2.68, 95% CI [1.41; 5.08], *p* = 0.0026) (Figure 3). The level of heterogeneity was moderate (I^2^ = 69%, τ^2^ = 0.354).

### 2.5. Overall Risk of Bias of CXCR4

Of the included five studies, three studies were rated with low risk of bias [22,46,50,51,56] and two with moderate risk of bias [36,48]. Therefore, the overall risk of bias was assessed as mainly low. Summed up, this systematic review and meta-analysis reveals CXCR4 as a meaningful prognostic biomarker.

## 3. Discussion

In summary, this systematic review and meta-analysis elucidate that tumoral overexpression of CXCR4 in 1381 patients represents a promising prognostic factor, resulting in significantly shortened overall survival as well as disease-free survival in colorectal cancer patients.

In the present systematic review and meta-analysis, tumoral overexpression of the chemokine receptor CXCR4 serves as a significant biomarker of poor prognosis in colorectal cancer patients. This could offer the opportunity of developing conceivably future targeted therapies with potential improvement of life expectancy.

Studies of CXCR4 included a total of 536 patients with CRC with a focus on overall survival and a total of 845 patients with CRC with a focus on disease-free survival. It was shown that both a significantly diminished overall survival as well as disease free survival could be demonstrated in patients with tumoral overexpression of CXCR4. Zengin et al. pointed out that CXR4 and CXCL12 overexpression in 260 colorectal cancer patients are also associated with a significantly worse prognosis regarding overall respectively disease-free survival [22]. Kim et al. examined six CRC cell lines and one hundred twenty-five CRC patients partly even matched with samples of synchronous liver metastasis [48]. Screening of samples and cell lines identified CXCR4 as the prominent chemokine receptor—an overexpression of CXCR4 in CRC tissue was confirmed with a significant negative impact on survival [48]. Kim et al. have a larger hazard ratio compared to the other studies, which may be attributed to the smaller number of patients [48]. Furthermore, Ottaiano et al. illustrated that overexpression of chemokine receptor CXCR4 was unfavorable for CRC patients. In their publication, seventy-two patients with stages II-III CRC were selected and operated on with curative intent [51]. Ottaiano et al. also examined the expression of CXCR4 in colon cancer cells using immunocytochemistry, and they came to a positive result in all cell lines examined. Patients with high CXCR4 expression revealed significantly worse disease-free survival compared to patients with low or no expression [51].

Moreover, Xu et al. were able to demonstrate that there is a correlation between clinicopathological characteristics, prognosis, and treatment of colorectal cancer. They analyzed the CXCR4 mRNA expression in colorectal carcinoma tissue from 48 patients using qRT-PCR and compared the expression level with the corresponding non-tumorous tissue [46]. Their data confirmed that an overexpression of CXCR4 is associated with worse overall survival [46].

In this systematic review, the chemokines CXCR7 and CXCL12 were also examined in more detail due to the acquainted interactions in the chemokine axis CXCR4–CXCL12-CXCR7. The CXCL12–CXCR4/CXCR7 axis plays an important role in the treatment strategy of patients with CRC [29]. D’Alterio et al. highlight that a concomitant negative or low expression of CXCR7, together with a high expression of CXCR4, is significantly associated with poor disease-free survival [36]. Further, high expression of CXCR4 together with negative or low expression of CXCL12 resulted in significantly worse relapse-free survival in the sixty-eight patients. The interaction of different chemokines may foster a negative impact on survival [36]. Both D’Alterio et al. and Kim et al. confirmed in their studies that high expression of chemokine CXCR4 represents a poor prognosis [36,48]. Zhang et al. confirm in their study that worse overall survival is associated with overexpression of CXCR4 in 125 samples from patients with stage II and III colon cancer [56]. Wang et al. also verified a worse prognosis regarding high CXCR4 expression in 388 patients suffering from colorectal cancer [50]. Regarding the quantitative results with respect to the overall risk of bias was assessed as low as can be seen in Table 2 and Table 3. Therefore, the results seem to be reliable.

Consequently, in our systematic review and meta-analysis, the random-effects model revealed a significantly diminished overall survival for CXCR4, pointing out CXCR4 as a prognostic factor.

Some limitations have to be mentioned in this systematic review and meta-analysis. The included studies did not all use the same detection methods for chemokine expression, e.g., qRT-PCR, immunohistochemistry or western blot. Further sources of clinical heterogeneity are follow-up periods of different lengths, some studies focused on special tumor stages, as well as studies were included independently of received neoadjuvant radio-/chemotherapy. Future studies regarding the influence of chemokines on survival should take these complex situations into account. Because only a small number of studies were included in the meta-analysis, the statistical heterogeneity may have been imprecisely estimated leading to too narrow confidence intervals of the pooled effects. A major strength of this systematic review and meta-analysis is the large number of 5556 screened studies focusing on chemokine tumoral expression in colorectal cancer patients. Moreover, this systematic review and meta-analysis focus on all chemokines that are known to be expressed in CRC tumor tissue in order to help identify promising new target options for tailored patient therapy approaches.

Immunotherapies are promising therapeutic approaches that can intervene in tumor growth using CXCR4 antibodies. There are some promising completed phase 1 clinical trials, such as NCT02695966 in pancreatic cancer and NCT02179970 in colorectal and pancreatic cancer targeting receptor CXCR4. In addition, there are other studies in phase 2, such as COMBAT/KEYNOTE-202-Study (NCT02826486) in metastatic pancreatic adenocarcinoma, NCT02907099 in metastatic pancreatic cancer and NCT03168139 in colorectal and pancreatic cancer, which are expected to have a promising result in a small cohort group [29]. The analysis of the data from the phase IIa study (NCT02826486) suggests that a combination of PD-1 blockade and CXCR4 antagonist can lead to an improvement in pancreatic ductal adenocarcinoma survival [68]. Besides, more clinical studies are being conducted focusing on antibodies against CCR2, CCR4, CCR5 and CXCR4 and nanobodies against CCL2, CCL5, CXCL11 and CXCL12 [6,69,70]. CXCR4 antagonists are considered to have an important function in chemotherapy due to the interference between tumor and stromal cells [71]. A study showed that the CXCR4 antagonist Plerixafor mobilizes leukemia cells, thereby leading to sensitization of cytotoxic therapy and improvement of hematopoietic cell transplantation (HCT) [72].

Kotb, R.M. et al. confirm that the chemokine receptor CXCR4 represents a starting point for optimizing the therapeutic outcome of trastuzumab in breast cancer patients [73].

Nevertheless, further studies and phase II/III trials are needed evaluating CXCR4 antagonists serving as potential new targeted therapies in colorectal cancer patients. Taken together, the chemokine receptor CXCR4 represents a promising future therapeutic target.

## 4. Materials and Methods

For this systematic review and meta-analysis, a literature search was performed according to the PRISMA guidelines (“Preferred Reporting Items for Systematic Reviews and Meta-Analyses”) [74]. The study was registered at PROSPERO, an international prospective register of systematic reviews, in 2020 CRD42020157312.

### 4.1. Systematic Literature Search

We conducted a systematic literature search in MEDLINE (via PubMed), Cochrane Central Register of Controlled Trials (CENTRAL) and Web of Science on 31 December 2023.

In MEDLINE (via Pubmed) was the search strategy the following: “Chemokines”[Mesh] OR chemokin*[tiab] OR CCL*[tiab] OR CXC*[tiab] OR IL-8[tiab] OR Interleukin-8[tiab] OR I-309[tiab] OR TCA-3[tiab] OR MCP-1[tiab] OR MIP-1α[tiab] OR MIP-1β[tiab] OR RANTES[tiab] OR C10[tiab] OR MRP-2[tiab] OR MARC[tiab] OR MCP-3[tiab] OR MCP-2[tiab] OR CCF18[tiab] OR MIP-1γ[tiab] OR Eotaxin*[tiab] OR MCP-5[tiab] OR MCP-4[tiab] OR NCC-1[tiab] OR Ckβ10[tiab] OR HCC-1[tiab] OR MCIF[tiab] OR Ckβ1[tiab] OR NCC-2[tiab] OR Leukotactin-1[tiab] OR MIP-5[tiab] OR HCC-2[tiab] OR NCC-3[tiab] OR LEC[tiab] OR NCC-4[tiab] OR LMC[tiab] OR Ckβ12[tiab] OR TARC[tiab] OR dendrokine*[tiab] OR ABCD-2[tiab] OR PARC[tiab] OR DC-CK1[tiab] OR AMAC-1[tiab] OR Ckβ7[tiab] OR MIP-4[tiab] OR ELC[tiab] OR Exodus-3[tiab] OR Ckβ11[tiab] OR Exodus-1[tiab] OR Ckβ4[tiab] OR SLC[tiab] OR 6Ckine[tiab] OR Exodus-2[tiab] OR Ckβ9[tiab] OR TCA-4[tiab] OR MDC[tiab] OR DC/β-CK[tiab] OR MPIF-1[tiab] OR Ckβ8[tiab] OR MIP-3[tiab] OR MPIF-1[tiab] OR Eotaxin-2[tiab] OR MPIF-2[tiab] OR Ckβ6[tiab] OR TECK[tiab] OR Ckβ15[tiab] OR Eotaxin-3[tiab] OR MIP-4α[tiab] OR IMAC[tiab] OR TSC-1[tiab] OR CTACK[tiab] OR ILC[tiab] OR eskine*[tiab] OR PESKY[tiab] OR skinkine[tiab] OR MEC[tiab] OR CXC*[tiab] OR Gro-α[tiab] OR GRO1[tiab] OR NAP-3[tiab] OR Gro-β[tiab] OR GRO2[tiab] OR MIP-2α[tiab] OR Gro-γ[tiab] OR GRO3[tiab] OR MIP-2β[tiab] OR PF-4[tiab] OR ENA-78[tiab] OR GCP-2[tiab] OR NAP-2[tiab] OR CTAPIII[tiab] OR β-Ta[tiab] OR PEP[tiab] OR NAP-1[tiab] OR MDNCF[tiab] OR GCP-1[tiab] OR MIG[tiab] OR CRG-10[tiab] OR IP-10[tiab] OR CRG-2[tiab] OR I-TAC[tiab] OR β-R1[tiab] OR IP-9[tiab] OR SDF-1[tiab] OR PBSF[tiab] OR BCA-1[tiab] OR BLC[tiab] OR BRAK[tiab] OR bolekine*[tiab] OR lungkine*[tiab] OR WECHE[tiab] OR SRPSOX[tiab] OR DMC[tiab] OR VCC-1[tiab] OR XCL1[tiab] OR lymphotactin*[tiab] OR SCM-1α[tiab] OR ATAC[tiab] OR SCM-1β[tiab] OR CX3CL1[tiab] OR fractalkine*[tiab] OR neurotactin*[tiab] OR ABCD-3[tiab])

AND

((colorectal[tiab] OR colon[tiab] OR colonic[tiab] OR rectum[tiab] OR rectal[tiab] OR CRC[tiab]) AND (cancer*[tiab] OR adenocarcinom*[tiab] OR carcinom*[tiab] OR neoplas*[tiab])) OR “Colorectal Neoplasms”[Mesh] NOT (animals [mh] NOT humans [mh])

### 4.2. Study Selection

Studies including patients with colorectal cancer, colon cancer or rectal cancer who underwent surgery with or without perioperative radiochemotherapy or chemotherapy alone were included. All publications that examined chemokine expression in tumor tissue with survival were included. In these studies, tumor tissue was compared with healthy colorectal mucosa tissue. Publications examining and analyzing chemokine expression in blood were excluded. Animal studies, editorials, letters, meeting abstracts and comments were also excluded, as well as all publications for which the full text was not available. Studies focused on colorectal metastases were excluded, too. There were restrictions regarding the language. Publications that were written in English, German and French were included; other languages were excluded due to lack of language skills. There were no restrictions regarding the publication year. The screening process and study selection from title and abstract were performed independently by two reviewers. Any disagreement concerning inclusion or exclusion was decided by consensus.

### 4.3. Data Extraction

The extraction of the data was performed independently by two reviewers. The following data were extracted: title, first author, publication year, location research, journal, language, trial registered, conflict of interest, funding, number of patients, duration of months choosing patients, duration of follow-up, TNM and grade. For each chemokine, the hazard ratio (HR) with 95% confidence interval (CI) limits for overall survival (OS) and disease-free survival (DFS) from univariable analysis were extracted.

If data from a Gene Expression Omnibus (GEO) database were used, it was checked whether the data came from patients or animal tissue. Only human data were extracted, and animal data were excluded. Care was also taken to ensure that the different datasets from the GEO database were only used once in the analysis in order to avoid the risk of bias.

### 4.4. Risk of Bias—Critical Appraisal

The Quality in Prognosis Studies (QUIPS) tool was used to evaluate the risk of bias and the quality of all the studies [75]. Two investigators (J.F.-H. and F.K.) assessed the methodological quality of the included studies. Study participation, attrition, prognostic factor measurement, outcome measurement, study confounding and statistical analysis and reporting are the six domains of the QUIPS tool. Each study was in these six domains rated as high risk, moderate risk or low risk. Most important among all domains is study confounding, which was then selected as the overall risk of bias. A low risk of bias was defined as no serious alteration of results. A moderate risk of bias was classified as a slightly serious outcome, and a high risk of bias was defined as a serious alteration of the results.

### 4.5. Data Handling and Statistical Analysis

The main outcome of this systematic review and meta-analysis was the overall survival. Data on cancer-specific survival (CSS) was equated to overall survival (OS). An additional outcome was disease-free survival. Progression-free survival, regression-free survival, relapse-free survival and recurrence-free survival were counted as disease-free survival (DFS). If studies split their observations into a training and a validation set, the effect measures based on the validation set were used. A meta-analysis was only carried out if there were three or more studies available.

The hazard ratio (HR) and the 95% confidence interval (CI) were used as effect measures for the survival endpoints. If a study did not explicitly report the HR and the 95% CI, they were estimated by other reported quantities using the formulas of Tierney et al. [76]. A *p*-value less than 0.05 was considered significant.

Statistical analysis were conducted using the software R (Version 4.3.1) and the package meta [77]. Random effects models were applied to account for the expected heterogeneity between the included studies. The between-study variance *τ^2^* and the *I^2^* statistic were used to assess the heterogeneity.

The results of the *I^2^* statistic were interpreted as follows: *I^2^* between 0% to 40% might not be important; 30% to 60% represents moderate heterogeneity; 50% to 90% represents significant heterogeneity; and 75% and above indicates significant heterogeneity [78].

In addition, to express the amount of heterogeneity, 95% prediction intervals were computed based on the t-distribution [79]. The prediction interval relates to the interval to predict the effect in a new study that is similar to the included studies in the meta-analysis.

The effect measures of the individual studies, as well as pooled results, were graphically visualized with forest plots. Statistical analysis of overexpression of chemokines was carried out using univariate values. The meta-analyses were performed using effect sizes that were estimated by univariable survival analyses in the individual studies.

## 5. Conclusions

In summary, in this systematic review and meta-analysis, a significant negative influence on overall as well as disease-free survival was shown for the chemokine receptor CXCR4 in primary colorectal cancer patients. Nevertheless, further studies are needed in order to confirm CXCR4 and its antagonists and to identify other chemokines and their receptors as prognostic factors and potential therapeutic targets.

## Figures and Tables

**Table 1:** Overview of the chemokine receptors with a focus on CXCR and corresponding ligands (adapted from [12]).

Chemokine Receptor	Chemokine Ligand
CXCR1	CXCL6, CXCL8
CXCR2	CXCL1, CXCL2, CXCL3, CXCL5, CXCL6, CXCL7, CXCL8
CXCR3	CXCL9, CXCL10, CXCL11, CXCL12
CXCR4	CXCL12
CXCR5	CXCL13
CXCR6	CXCL16
CXCR7	CXCL11, CXCL12

**Table 2:** Overview of all included studies in this systematic review and meta-analysis evaluated by the Quality in Prognosis Studies (QUIPS) tool. OS = overall survival, DFS = disease-free survival.

Study	Number of Patients	SurvivalType	Investigated Chemokine	Study Participation	Study Attrition	Prognostic Factor Measurement	Outcome Measurement	Study Confounding	Statistical Analysis and Reporting
Ruan G. T. 2019 [39]	212	OS	CXCL3	Low	Low	Low	Low	Low	Low
Zengin, M. 2020 [22]	260	OS DFS	CXCR4 CXCL12	Low	Low	Low	Low	Low	Moderate
Li, X. 2019 [40]	491	OS DFS	CXCL9 CXCL10 CXCL11 CXCL13	Moderate	Low	Low	Low	Low	Low
Gong, Y.Z. 2021 [41]	438	OS	CXCL1	Moderate	Low	Low	Low	Moderate	Moderate
Li, L. 2021 [42]	160	OS	CXCL7	Low	Low	Low	Low	Moderate	Low
Liu, M.J. 2021 [43]	342	OS	CXCL11	Low	Low	Low	Low	Low	Low
Chen, Z. 2019 [44]	142	OS	CXCL16	Low	Low	Low	Low	Low	Low
Okikawa, S. 2021 [45]	98	OS DFS	CXCL12	Low	Moderate	Low	Moderate	Low	Moderate
Xu, C. 2018 [46]	48	OS	CXCR4	Low	Moderate	Low	Low	Low	Low
Luo, X. 2022 [47]	232	OS	CXCL1 CXCL2 CXCL8 CXCL13 CXCL14	Low	Low	Low	Low	Moderate	Moderate
Kim, J. 2005 [48]	92	OS DFS	CXCR4	Low	Low	Low	Moderate	Moderate	Moderate
Terada, H. 2005 [49]	87	OS	CXCL8	Low	Moderate	Low	Low	Low	Low
Wang, S. 2010 [50]	388	DFS	CXCR4	Low	Low	Low	Low	Low	Low
Ottaiano, A. 2006 [51]	72	DFS	CXCR4	Low	Low	Low	Low	Low	Low
Watanabe, H. 2008 [52]	101	OS	CCL2	Low	Low	Low	Low	Moderate	Moderate
Akishima-Fukasawa, 2009 [53]	165	OS DFS	CXCL12	Low	Low	Low	Low	Low	Low
Oladipo, O. 2011 [54]	228	OS DFS	CXCL8	Low	Low	Low	Low	Low	Low
Wu, Z. 2012 [55]	112	OS	CXCR3	Low	Low	Low	Low	Low	Low
Zhang, N.H. 2012 [56]	125	OS	CXCR4	Low	Low	Low	Low	Low	Low
Yuan, R. 2013 [57]	371	OS DFS	CCL18	Low	Low	Low	Low	Low	Low
Zou, Y. 2013 [58]	143	OS DFS	CCL21	Low	Low	Low	Low	Low	Low
Yang, D. 2015 [59]	96	OS DFS	CXCR7	Low	Low	Low	Low	Low	Low
Wu, Z. 2016 [60]	130	OS	CXCL9	Low	Low	Low	Low	Low	Low
Yao, H. 2020 [61]	101	OS	CXCR8 CXCL17	Low	Low	Low	Low	Low	Low
D’Alterio, 2014 [36]	68	OS DFS	CXCR4 CXCR7 CXCL12	Low	Moderate	Low	Low	Moderate	Low
Zeng, J. 2013 [62]	226	OS DFS	CXCL14	Low	Moderate	Low	Moderate	Moderate	Moderate
Lin, K. 2014 [63]	40	OS	CXCL14	Moderate	Moderate	Low	Low	Moderate	Moderate
Zhu, Y.X. 2014 [64]	136	OS DFS	CCX-CKR	Low	Low	Low	Low	Low	Low
Zhao, J. 2017 [65]	56	OS DFS	CXCR2	Low	Moderate	Low	Low	Low	Low
Zhuo, C. 2018 [66]	276	OS DFS	CXCL1	Low	Moderate	Low	Low	Low	Low
Li, R. 2023 [67]	643	OS	CXCL8	Low	Low	Low	Low	Moderate	Low

**Table 3:** Study characteristics: CXCR4—OS, DFS.

Study Information	Evaluation of Cxcl12 and Cxcr4 to Predict Poor Survival in Lymph Node-Positive Colorectal Cancer Patients	CXCR4 Overexpression Is Correlated with Poor Prognosis in Colorectal Cancer	Chemokine Receptor CXCR4 Expression in Colorectal Cancer Patients Increases the Risk for Recurrence and for Poor Survival	Co-Expression of CXCR4 and CD133 Proteins Is Associated with Poor Prognosis in Stage II-III Colon Cancer Patients	A Prognostic Model Comprising pT Stage, N Status, and the Chemokine Receptors CXCR4 and CXCR7 Powerfully Predicts Outcome in Neoadjuvant Resistant Rectal Cancer Patients	Nuclear Expression of CXCR4 Is Associated with Advanced Colorectal Cancer	Overexpression of both CXC Chemokine Receptor 4 and Vascular Endothelial Growth Factor Proteins Predicts Early Distant Relapse in Stage II–III Colorectal Cancer Patients
First Author	Zengin, M.	Xu, C.	Kim, J.	Zhang, N.H.	D’Alterio, C.	Wang, S.	Ottaiano, A
Year Publication	2020	2018	2005	2012	2014	2010	2006
Country	Turkey	China	USA	China	Italy	Taiwan	Italy
Number of patients	260	48	92	125	68	388	72
AJCC	- AJCC 3: 161 - AJCC 4: 99	Not available	Primary AJCC stages: - AJCC 1: 29 - AJCC 2: 28 -AJCC 4: 35	- AJCC 2: 61 - AJCC 3: 64	Preoperative AJCC stage: - AJCC2: 9 - AJCC3: 59 Postoperative AJCC stage: - AJCC1: 29 - AJCC 2: 12 - AJCC 3: 27	- AJCC 1: 52 - AJCC 2: 133 - AJCC 3: 123 - AJCC 4: 80	- AJCC 2: 39 - AJCC 3: 33

## Data Availability

Extracted Data are available upon request.

## Supplementary Materials

The following supporting information can be downloaded at https://www.mdpi.com/article/10.3390/ijms25105374/s1, Figure S1A: CXCL1—OS; Figure S1B: CXCL8—OS; Figure S1C: CXCL12—OS; Figure S1D: CXCL12—DFS; Figure S1E: CXCL14—OS; Table S1A: CXCL1—OS; Table S1B: CXCL8—OS; Table S1C: CXCL12—OS, DFS; Table S1D: CXCL14—OS.
