# Supplementary material for: Chemokines as Prognostic Factor in Colorectal Cancer Patients: A Systematic Review and Meta-Analysis
Source: Int J Mol Sci. 2024 May 15;25(10):5374. doi: 10.3390/ijms25105374 (PMC11121014; doi:10.3390/ijms25105374)

Figure 1A: CXCL1 – OS

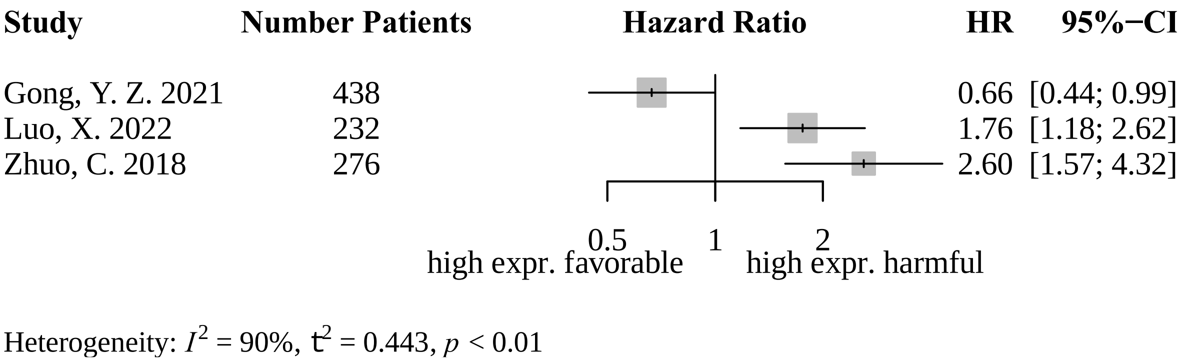

Figure 1B: CXCL8 – OS

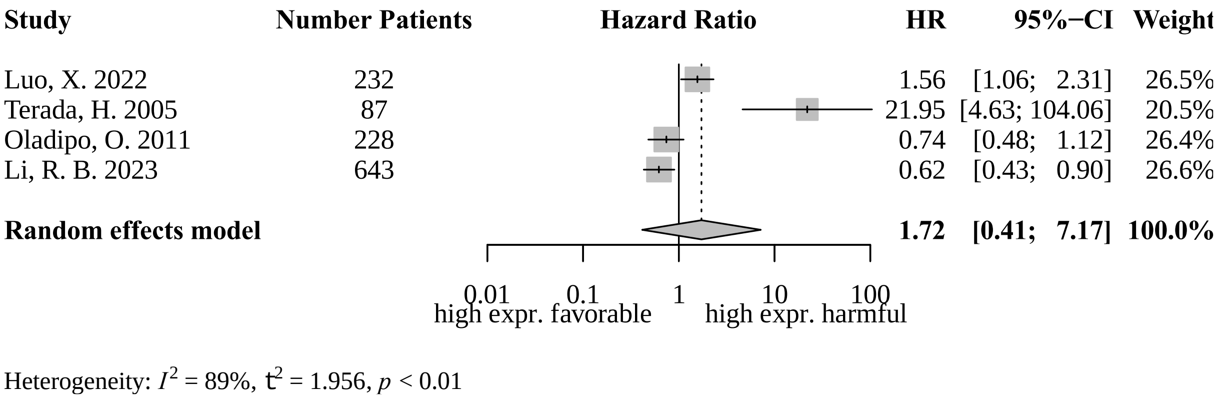

Figure 1C: CXCL12 – OS

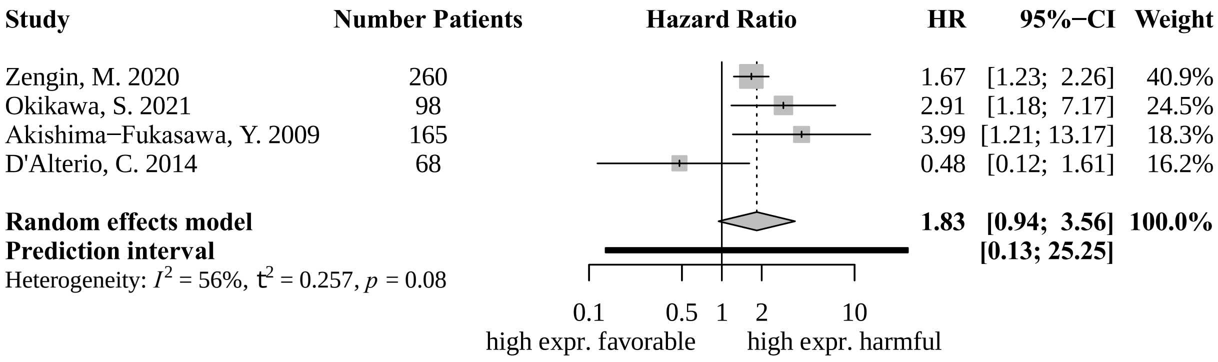

Figure 1D: CXCL12 – DFS

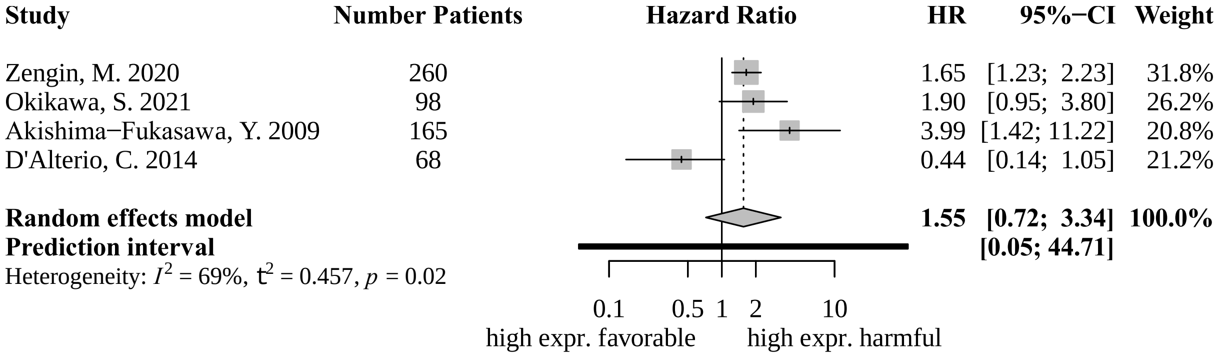

Figure 1E: CXCL14 – OS

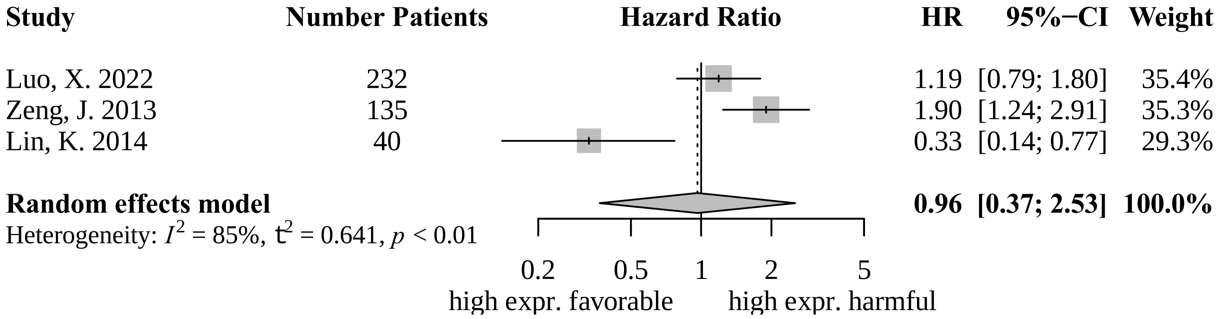

Supplement: Supplementary file 1 [file ijms-25-05374-s001.zip › Supplementary_Figure_Minorrev.pdf]
