# Supplementary material for: Chemokines as Prognostic Factor in Colorectal Cancer Patients: A Systematic Review and Meta-Analysis
Source: Int J Mol Sci. 2024 May 15;25(10):5374. doi: 10.3390/ijms25105374 (PMC11121014; doi:10.3390/ijms25105374)
Supplement: Supplementary file 1 [file ijms-25-05374-s001.zip › Supplementary_Table_Minorrev.pdf]

Supplement - Tables

Table 1A:

CXCL1 – OS

**Study information**	**Diagnosis and prognostic value of C-X-C motif chemokine ligand 1 in colon adenocarcinoma based on The Cancer Genome Atlas and Guangxi cohort**	**Associations of C-X-C motif chemokine ligands 1/2/8/13/14 with clinicopathological features and survival profile in patients with colorectal cancer**	**Chemokine (C-X-C motif) ligand 1 is associated with tumor progression and poor prognosis in patients with colorectal cancer**
First Author	Gong,Y.Z.	Luo, X.	Zhuo, C.
Year Publication	2021	2022	2018
Country	China	China	China
Journal	Journal of Cancer	Oncology letters	Bioscience Reports
Number of patients	438	232	276
AJCC	- AJCC 1: 73<br>- AJCC 2: 167<br>- AJCC 3: 126<br>- AJCC 4: 61<br>(information was not obtained in 11 patients)	- AJCC 1: 30<br>- AJCC 2: 109<br>- AJCC 3: 93	- AJCC 1: 24<br>- AJCC 2: 90<br>- AJCC 3: 120<br>- AJCC 4: 42

Table 1B:

CXCL8 – OS

**Study information**	**Associations of C-X-C motif chemokine ligands 1/2/8/13/14 with clinicopathological features and survival profile in patients with colorectal cancer**	**Association of interleukin-8 and plasminogen activator system in the progression of colorectal cancer**	**The expression and prognostic impact of CXC-chemokines in stage II and III colorectal cancer epithelial and stromal tissue**	**Identification and validation of a pyroptosis-related prognostic model for colorectal cancer**
First Author	Luo, X.	Terada, H.	Oladipo, O.	Li, R. B.
Year Publication	2022	2005	2011	2023
Country	China	Japan	Northern Ireland	China
Journal	Oncology letters	European Surgical Research	British Journal of Cancer	Functional & Integrative Genomics
Number of patients	232	87	228	643
AJCC	- AJCC 1: 30<br>- AJCC 2: 109<br>- AJCC 3: 93	Not available	- AJCC 2: 146<br>- AJCC 3: 82	Not available

Table 1C:

CXCL12 – OS, DFS

**Study information**	**Evaluation of Cxcl12 and Cxcr4 to Predict Poor Survival in Lymph Node-Positive Colorectal Cancer Patients**	**SDF-1 expression after preoperative chemoradiotherapy is associated with prognosis in patients with advanced lower rectal cancer**	**Prognostic significance of CXCL12 expression in patients with colorectal carcinoma**	**A prognostic model comprising pT stage, N status, and the chemokine receptors CXCR4 and CXCR7 powerfully predicts outcome in neoadjuvant resistant rectal cancer patients**
First Author	Zengin, M.	Okikawa, S.	Akishima-Fukasawa, Y.	D'Alterio, C.
Year Publication	2020	2021	2009	2014
Country	Turkey	Japan	Japan	Italy
Journal	Polish Journal of Pathology	The Journal of Medical Investigation	Anatomic Pathology	International Journal of Cancer
Number of patients	260	98	165	68
AJCC	- AJCC 3: 161<br>- AJCC 4: 99	- AJCC 0: 11<br>- AJCC 1: 30<br>- AJCC 2: 27<br>- AJCC 3: 24<br>- AJCC 4: 6	- AJCC 2: 72<br>- AJCC 3: 93	Preoperative AJCC stage:<br>- AJCC 2: 9<br>- AJCC 3: 59<br><br>Postoperative AJCC stage:<br>- AJCC 1: 29<br>- AJCC 2: 12<br>- AJCC 3: 27

Table 1D:

CXCL14 - OS

**Study information**	**Associations of C-X-C motif chemokine ligands 1/2/8/13/14 with clinicopathological features and survival profile in patients with colorectal cancer**	**Chemokine CXCL14 is associated with prognosis in patients with colorectal carcinoma after curative resection**	**Expression and effect of CXCL14 in colorectal carcinoma**
First Author	Luo, X.	Zeng, J.	Lin, K.
Year Publication	2022	2013	2014
Country	China	China	China
Journal	Oncology letters	Journal of Translational Medicine	Molecular Medicine Reports
Number of patients	232	226	40
AJCC	- AJCC 1: 30<br>- AJCC 2: 109<br>- AJCC 3: 93	- AJCC 1: 8<br>- AJCC 2: 83<br>- AJCC 3: 127<br>- AJCC 4: 8	- AJCC 1-2: 18<br>- AJCC 3-4: 22
